# Exploring the Role of Acacia (*Acacia seyal*) and Cactus (*Opuntia ficus-indica*) Gums on the Dough Performance and Quality Attributes of Breads and Cakes

**DOI:** 10.3390/foods11091208

**Published:** 2022-04-21

**Authors:** Shahzad Hussain, Mohamed Saleh Alamri, Abdellatif A. Mohamed, Mohamed A. Ibraheem, Akram A. Abdo Qasem, Ghalia Shamlan, Ibrahim A. Ababtain

**Affiliations:** Department of Food Science and Nutrition, King Saud University, Riyadh 1145, Saudi Arabia; msalamri@ksu.edu.sa (M.S.A.); abdmohamed@ksu.edu.sa (A.A.M.); mfadol@ksu.edu.sa (M.A.I.); aqasem@ksu.edu.sa (A.A.A.Q.); shamlana@ksu.edu.sa (G.S.); ababtain.ibr@gmail.com (I.A.A.)

**Keywords:** cactus gum, acacia gum, flour, bread, cake, sensory, rheology

## Abstract

Two hydrocolloids, acacia gum and cactus gum, were tested in the current study to see if they could improve the quality of the dough or have an effect on the shelf life of pan bread and sponge cake. Both gums considerably (*p* < 0.05) enhanced the dough development time, softness, and mixing tolerance index while decreasing the water absorption. Although the dough was more stable with the addition of acacia gum than with cactus gum, the control sample had the highest peak, final, breakdown, and setback viscosities. Acacia gum, on the other hand, resulted in a higher wheat-flour-slurry pasting temperature (84.07 °C) than cactus gum (68.53 °C). The inclusion of both gums, particularly 3%, reduces the gel’s textural hardness, gumminess, chewiness, springiness, and adhesiveness. Lightness (L*) and yellowness (b*) were both increased by the addition of acacia gum to bread and cake, whereas the addition of cactus gum increased both color parameters for cakes. The use of acacia gum increased the bread and cake’s volume. Cactus gum, on the other hand, caused a decrease in bread hardness after 24 h and 96 h. The cake containing acacia gum, on the other hand, was the least stiff after both storage times. Similarly, sensory attributes such as the crumb color and overall acceptability of the bread and cake were improved by 3% with acacia gum. For these and other reasons, the addition of cactus and acacia gums to bread and cake increased their organoleptic qualities, controlled staining, and made them softer.

## 1. Introduction

Bread is thought to be one of the oldest manmade foods. It is regarded as an important carbohydrate source in the food pyramid because it is consumed in many parts of the world. It is typically prepared by baking the dough or, in some cases, steaming the dough (mantou). Bread dough is made up of flour, usually wheat flour, water, and a leavening agent [1]. Bread can be leavened in a variety of ways, including the use of industrial yeast (*Saccharomyces cerevisiae*), chemicals (baking soda), naturally occurring microbes (lactic acid bacteria), or high-pressure aeration, which produces gas bubbles that fluff up the bread. In addition, commercial bread usually contains food additives to enhance the color, flavor, nutrition, and texture and to prolong the shelf life [2].

Cake is a sweet bakery product with a distinct texture and flavor. Flour, eggs, sugar, and oil are the major ingredients. It is one of the most popular sweet bakery items in the world and is enjoyed in large quantities by people of all ages [3,4]. It has a porous texture and a spongy hollow with a thin wall. The porosity is mostly caused by food additives that produce carbonic acid, carbon dioxide, and air circulation via heating oil, eggs, and sugar. The growth of the gluten network creates a spongey hollow, which keeps the structure from collapsing [4]. According to Jia et al. [5], a cake can be good or bad based on its viscosity, its specific gravity, and how the bubbles are spread.

Gum Arabic (acacia), for example, is a hydrocolloid that is widely used as a bread addition. It is used in bread to improve softness, extend shelf life by managing the moisture content and preventing retrogradation, modify texture, and raise specific volume [6,7]. Furthermore, the effect of hydrocolloid addition on bread quality is influenced by the amount and type of hydrocolloid used [8]. Numerous studies have been conducted to determine the impact of gums on bread quality. Several gums, including xanthan, carrageenan, sodium alginate, and gum Arabic, decreased the hardness and springiness retention of steamed potato bread during storage [8]. According to Sharadanant and Khan [9], adding gum Arabic to bread enhances the volume of the loaf while also improving the softness, cell wall structure, and texture of the bread. According to Aboulnaga et al. [10], the physical parameters (specific volume, weight, and loaf volume) of pan bread were greatly improved by the addition of different gums (Arabic, guar, xanthan). The addition of locust bean gum, xanthan gum, guar gum, or carboxymethyl cellulose (CMC) to wheat bread slowed theft and increased the bread’s quality [11]. Gum Arabic, xanthan gum, CMC, and other gums are commonly used in cake formulations to perform a variety of functions, including maintaining the cake’s moisture content, preventing starch retrogradation (staling), increasing the softness of the cake (which is related to porosity and volume), improving the mouthfeel (which is ultimately related to consumer acceptance), and increasing and maintaining the cake batter’s stability [12,13,14]. According to Salehi and Kashaninejad [15], the influence of gums on final cake volume is mostly attributable to an increase in batter viscosity, which slows the diffusion of gas and traps it during the early stages of baking. The impact of gums on cake making has been researched by a number of writers. Turabi et al. [16] found that adding guar gum, xanthan, carrageenan, or locust bean gum to microwave-baked rice cakes increased the number of holes. According to Noorlaila, Hasanah, Asmeda, and Yusoff [13], sponge cake with hydroxypropylmethylcellulose exhibited less hardness during storage than sponge cake with xanthan gum.

Cactus mucilage is a slimy substance (polysaccharides) that appears shortly after the cuticle of cladodes is broken or cut. Cladodes is a member of the Cactaceae family, which includes modified cactus stems with huge spines and small hairs that resemble thrones [17,18]. Cactus mucilage has a high molecular weight of roughly 3.67 × 106 g/mol and is well-known for its hydrophilic nature, which allows it to effectively bind to water [19,20]. Due to their safety, biocompatibility, and availability, plant-based viscous polysaccharides (mucilage or gums) are becoming increasingly popular in food items. According to Dick et al. [21], because of its hydrocolloid properties, cactus mucilage can be utilized as an ingredient to improve the texture of food products. Cactus mucilage has been recommended as an emulsifying agent by Manhivi et al. [22], and De Waal et al. [23] claimed that it can also be utilized as a gelatin substitute in Turkish delight. The goal of this study was to see how locally extracted acacia and cactus gums affected the rheology and the textural and functional aspects of flour blends, as well as the baking performance of such blends in cakes and breads.

## 2. Materials and Methods

### 2.1. Isolation of Gums

Fresh cactus cladodes and acacia gum exudates were collected from the Dirab Agriculture Research Station of King Saud University (Riyadh, Saudi Arabia). Acacia gum (AG) and cactus gum (CG) powders were prepared according to the methods described in our previous publication [24]. The proximate composition of CG was recorded as (moisture, 8.5%; protein, 5.1%; total carbohydrates, 51.20%) whereas for AG (moisture, 8.10%; protein 2.12%; and total carbohydrates, 85.12%).

### 2.2. Preparation of Wheat Flour-Gum Mixtures

Flour and gum mixtures were made by substituting AG and CG powders for 1 and 3% of the wheat flour in the combination. The blended flours were stored in sealed jars until they were needed for analyses and baking. All the analyses and baking occurred within a week of the preparation of the blends.

### 2.3. Rapid Visco Analyzer Measurements (RVA)

Tests on the pasting ability of the flour combinations were carried out using a Rapid Visco Analyzer (Newport Scientific, Sydney, Australia). The sample (3.5 g) at 14% adjusted moisture content was placed in RVA canisters and then distilled water was added until the canisters achieved a total weight of 28.5 g. The slurry was kept at 50 °C for 50 s, heated to 95 °C at a rate of 12.16 °C/min and held for 5 min, and then cooled to 50 °C in 2 min and held for another 2 min. The data were analyzed with the use of the thermocline window program, which was provided by the manufacturer [25].

### 2.4. Gel Texture

To obtain the texture of flour gels obtained from RVA canisters, the gels were transferred to a 25 mL beaker and kept at room temperature (25 °C) overnight. The gels were tested using textural profile analysis test (TPA) at a speed of 0.5 mm/s and a distance of 10 mm using a Brookfield CT3 Texture Analyzer (Brookfield Engineering Laboratories, Inc., Middleboro, MA, USA) equipped with a 12.7 mm broad and 35 mm long cylindrical probe. Hardness, springiness, cohesiveness, and adhesiveness of the gel were all measured. Chewiness was calculated as a product of gumminess and springiness, whereas gumminess was computed as a product of hardness and cohesiveness [25].

### 2.5. Dough-Mixing Properties Using Doughlab

The micro-dough lab (Perten Instruments, Sydney, Australia) was used to determine the optimal water absorption capacity, reaching a peak of 500 farinograph (FU), utilizing a 4.00 ± 0.001 g sample at a 14% moisture basis. The weights of the samples and water were adjusted based on their moisture content. For 20 min, samples were mixed at a speed of 63 rpm and a temperature of 30 °C. For each sample, measurements were carried out at least three times in total. Following the creation of a mixing curve, the data were processed to determine the dough development time (min), stability time (min), softening time (FU), mixing tolerance index (FU), and quality number [26].

### 2.6. Bread-Baking Procedure

The AACC [27] method No. 10-09 was used to evaluate the baking performance of the control flour and the flour mixed with gum powder. A total of 100 g of flour was used, along with 3 g of instant dry yeast, 6 g of sugar, 0.02 g of improver, 4 g of nonfat instant milk powder, 1.5 teaspoons of sodium bicarbonate (salt), and 5 g of shortening. The amount of water used to make the dough was adjusted based on the amount of water absorbed by each individual blend (provided in Table 1), and the dough was mixed (based on development time, Table 1) in a blender (Kitchen Aid, KSM9, Michigan, MI, USA). The first proofing of the dough took 60 min, and the second proofing took another 30 min after punching. Following the second proofing, the dough was sheeted and molded, and allowed to rest for another 30 min before baking. All the bread loaves were baked for 20 min at 220 °C in the oven (National MFG. Co., Lincoln, NE 68508, USA). Following baking, the loaves were allowed to cool (4 h) before being sliced and stored for further testing.

### 2.7. Cake-Baking Procedure

The baking of the cakes was carried out in accordance with AACC [27] method No. 10-90. The ingredients for the cake are 100 g flour (control or with gum powder), 140 g sugar, 50 g shortening, 12 g nonfat dry milk, 210 g fresh eggs, 3 g baking powder, and water. After thoroughly mixing all the ingredients according to the instructions, the baking was completed at 190 °C. The baked cakes were allowed to cool (4 h) and then were stored in zip lock polythene bags at room temperature for texture studies for 4 days.

### 2.8. Crumb Color of Bread and Cakes

Color values such as L* (Lightness), a* (Greenness), and b* (Yellowness) were determined for bread and cake crumb samples using a Minolta color-grader equipped with a D65 light source [28].

### 2.9. Bread and Cake Firmness

Bread samples were kept at room temperature, and firmness was measured on days 2 and 4 using two central slices (25 mm thick) according to AACC [27] method 74-09. The TA-TXT texture analyzer was equipped with a 20 mm cylindrical probe. A 25% compression of a 25 mm thick sample resulted in a 6.25 mm compression distance, at which point the compression force value (CFV) of firmness was used and the data were processed using Exponent software. The cake samples were also compressed using the same probe at 25% strain on a 60 mm cake piece from the center. The probe was held at this distance for 60 s before being withdrawn from the sample and returned to its starting position, and the springiness percentage was calculated. For both tested products, the pretest and test speeds were kept constant at 1 mm/s.

### 2.10. Sensory Evaluation of Baked Products

A trained panel of judges (10) assessed the sensory qualities of bread and cake samples on a 9-point hedonic scale. Bread samples were evaluated for volume, taste, texture, crumb color, and overall acceptability, whereas cake samples were evaluated for taste, texture, crumb color, porosity, and overall acceptability.

### 2.11. Statistical Analyses

All measurements were carried out in triplicate. One-way analysis of variance was used to look at the data. Duncan’s Multiple Range (DMR) test at a *p*-value of 0.05 was used to compare means in PASW^®^ Statistics 18 software.

## 3. Results and Discussion

### 3.1. Dough Properties of Flour-Gum Mixtures

Dough is a gluten network formed by hydration, in which starch and other flour fractions are incorporated [29]. The dough properties are critical in achieving the desired organoleptic properties in the baked product. Water absorption, development time, stability, tolerance index, and quality number were estimated for the dough. Water absorption is the amount of water required to develop the dough’s consistency to 500 FU, and it varies depending on the amount of wheat gluten in the flour. However, the addition of non-protein fractions alters the absorption behavior of flour during dough development. The wheat flour with no gum had the highest water absorption of 61.47%, which was reduced to 59.93% with the addition of 1% acacia gum (Table 1). However, increasing the concentration of gum from 1% to 3% for both gums resulted in decreased water absorption, with the wheat flour containing the 3% acacia gum absorbing the least. This decrease in water absorption is expected due to the dilution of glutenin fractions, which resulted in poor dough network formation [30]. Chen et al. [31] discovered that glutenin, rather than glutelin, plays a critical role in water absorption and retention in the baked bread crumb structure. The dough absorbed less water when *Cordia myxa* (L.) fruits were added [32].

The dough development time (DDT) is the amount of time it takes for the flour dough to reach its maximum consistency while being mixed under shear. The DDT for the control wheat flour dough was the shortest (1.6 min), increasing to 3.9 min in the presence of cactus gum. The wheat flour dough with acacia gum, on the other hand, required nearly three times as long to develop the dough network. Surprisingly, increasing the acacia and cactus gum concentrations from 1% to 3% did not result in a significant change in the DDT. This delayed dough development could be attributed to the inhibited hydration of glutelin and glutenin fractions to develop gluten, as well as the added acacia gum competing with proteins for water. Huang et al. [33] reported an increase in DDT by incorporating water-soluble-resistant dextrins at various levels. A reduced DDT has been reported for dough containing ziziphus gums [32].

Dough stability refers to the dough’s mechanical strength and ability to maintain consistency over time. When acacia gum was added at 3%, the dough produced the most stable network. Wheat flour blended with cactus gum, on the other hand, produced the least stable dough. This suggests that the presence of acacia gum synergistically increased the strength of the gluten network. When acacia gum was replaced with cactus gum, the dough stability was reduced by half. These findings were supported by Alamri, Mohamed, Hussain, Ibraheem, Qasem, Shamlan, Hakeem, and Ababtain [32], who discovered that the addition of hydrocolloids from cordia and ziziphus reduced dough stability. The difference between the FU units at the highest consistency of the mixogram curve (at peak time) and the value after 5 min of mixing is used to calculate dough MTI. This is also an indirect indicator of dough softening. In general, lower MTI dough values, i.e., 30 FU or less, are considered very good for bread preparation. Higher MTI values (50 FU or higher) are more likely to result in poor dough handling properties and lower mechanical strength. The dough containing cactus gum had the highest MTI (111 FU), as evidenced by its 3% cactus gum content. The dough containing acacia, on the other hand, showed a 50% reduction in MTI when compared to the other samples, with the control dough containing no gum having the lowest MTI of 35.67 FU. As a result, the controlled dough presented the greatest resistance to shear mixing. The lower dough MTI indicates that the presence of acacia contributed positively to dough stability and provided a relatively stronger dough than its cactus gum counterparts. However, both gums were unable to maintain dough strength due to higher MTI values than the control. According to Alamri, Mohamed, Hussain, Ibraheem, Qasem, Shamlan, Hakeem, and Ababtain [32], the addition of ziziphus gum reduced dough resistance and increased MTI values.

Dough softening is also an indirect indicator of dough stability. The control wheat flour dough resulted in the least amount of dough softening, with only 91.67 FU of softening observed at the maximum dough consistency. The addition of both the acacia and cactus gums, on the other hand, caused the dough to soften, with the cactus gum having the highest softness of 146.63 FU at 3%. Although the presence of acacia resulted in increased dough softening, the presence of 1% acacia resulted in a higher degree of dough softening than the presence of 3% acacia. In general, the MTI and softening are linked, so as the MTI rises, the dough softens [34]. The distance (in millimeters) between the point of water addition in the flour and the point on the mixogram where the central line loses 20 FU can be seen along the time coordinate. This demonstrates the resistance to kneading and the stability of the dough. The control dough, which had no added gum, had the highest quality number of 61.23. However, when cactus gum was added at a higher concentration, the lowest quality number (40.97) was observed among all dough samples (3%). This decrease in the quality number could be due to gum–gluten interaction rather than gluten–gluten interaction in the control dough [32].

### 3.2. Pasting Properties of Flour–Gum Mixtures

**Figure 1:**
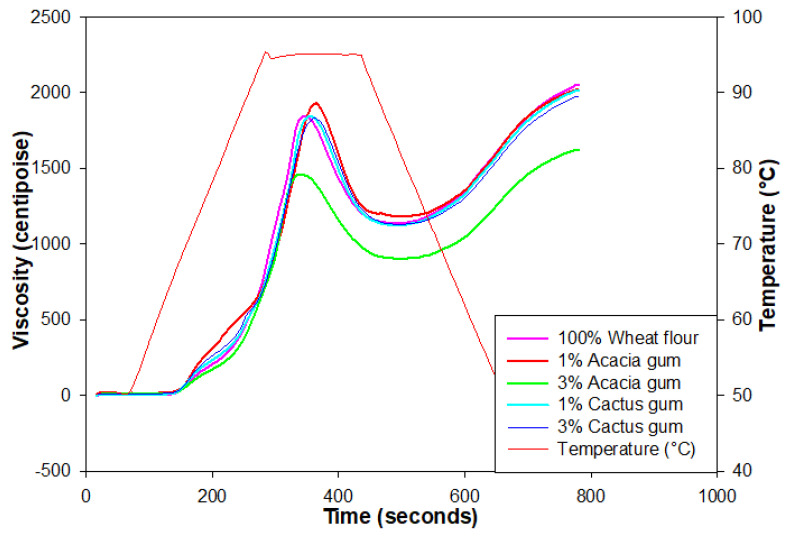
RVA profiles of wheat flour blends with acacia and cactus gums.

The wheat flour and gum mixes were tested using a rapid visco-analyzer to determine their pasting properties. In the presence of water, starch granules in the flour swelled to the maximum possible extent when heated and sheared in the presence of water [35]. Data corresponding to the pasting behavior of different blends are presented in Table 2, and Figure 1 displays the pasting profiles of the blends. Wheat flour, when used as a control, had the greatest PV (1886 cP), but the value dropped when gums were added. As the gum concentration in the mixes grew, so did the fall in the PV. For example, when the amount of gum-acacia was increased to 3%, the PV of the wheat flour was reduced from 1693 to 1453 cP. However, the PV value of the wheat flour was not significantly different from the cactus-containing samples, especially at lower levels of addition, when compared to gum acacia. Since the cactus had no effect on the swelling and leaching of amylose, acacia gum was the better choice. This decrease in PV is also a sign that the total starch content in the blends was diluted. The rise or fall in the PV of wheat flour gels was determined by the type and amount of gum–flour interaction. In the presence of AG or CG gums, which coated the starch granules and prevented their swelling when heated, the reduced PV could be the result of the reduced swelling of the granules [36]. The addition of ziziphus gum in the wheat flour lowered the PV by controlling the swelling of the wheat starch [32].

Disruptions and reductions in the viscosity of starch granules are both indicators of the paste’s stability under heat and shear, and BD is an indirect measure of this. As a result of heat under shear, the viscosity decreases following its peak. The BD significantly decreased when gums, such as acacia or cactus, were present, following the PV pattern. The control paste of wheat flour had the highest BD (736 cP), which decreased to 702 cP when cactus gum was added to the blend. An increasing starch breakdown value indicates poor resistance to shear and heat application. Acacia gum was found to be the most effective in reducing BD. The lower BD of the blends may have been due to the dilution of the overall starch content in the blend. It is possible that acacia gum stopped starch granules from being dissolved, which would have prevented crumbling and preserved the paste structure, but this is unlikely. In addition, previous research has shown that gums can reduce the risk of developing BD. According to Alamri et al. [32], adding gum Cordia to wheat flour paste decreased the BD and raised the system’s tolerance. The reduced swelling of the granules in the presence of gums may be the cause of the lower BD.

During the 50 °C holding phase, the final viscosity (FV) is the final viscosity of the dispersion of wheat flour and gum mixes. Leached amylose and small-molecular-weight amylopectin fractions in the system are the primary contributors to the FV. A similar trend to that seen in the PV data was seen in the FV data. The maximum FV (2056 cP) was found in the control wheat flour, whereas the lowest FV (1593 cP) was found in the wheat flour mix with three percent acacia gum. Gum blends with low-viscosity flours are available and suitable for food applications such as protective coatings, weaning foods, and confectionary products [37]. When starch is replaced with gum, the final viscosity decreases because there is less starch in the blend [38]. Similarly, ziziphus gum reduced the viscosity of wheat flour, as reported in the literature [32]. A decrease in FV was also seen when gum arabic was added to cassava and corn starches.

When the paste cools down, the amylose chains begin to align, and recrystallization begins, the SB is gained. The short-term cooling of wheat flour pastes from 95 to 50 °C results in a change in the FV and BD in the RVA profile. The wheat flour with cactus gum was found to cause the greatest setback, followed by the wheat flour with acacia gum, and, finally, the control wheat flour. Wheat starch with more gum in it has a lower SB than starch with less gum. A decreased setback in cooling was seen when gums were added, especially at greater concentrations, to wheat flour in order to reduce the retrogradation of starch. A probable reduction in the SB could be attributed to the disfavored amylose–amylose interaction in the presence of gums, in which the gums serve as a barrier between the leached amylose and govern their lining and network creation, respectively. Overall, the SB trend was nearly identical to the FV trend, with a higher FV resulting in a higher overall SB. Both Alamri et al. [32], and Kiprop et al. [39] found that adding gum Arabic or gum Cordia decreased the SB, which lends credence to this study.

The viscosity of wheat flour dispersions begins to rise at PT when swelling and simultaneous starch rupture are both triggered by heat and shear [35]. This serves as a gauge of the amount of energy required to transform starch into gelatin. The blends of wheat flour and acacia gum at 3%, followed by the blend with 1% acacia, had the highest PT of 84.0 °C. According to this, wheat flour blended with acacia gum necessitates a greater gelatinization temperature and a longer baking time than normal wheat flour. Acacia gum enhanced the flour thermal behavior by competing for water and interfering with wheat flour starch absorption. The rise in the PT indicates that the starch in the flour was not gelatinized as quickly as it should have been during baking, which may have contributed to the bread’s higher volume. Cactus gum, however, resulted in a decrease in the PT of wheat flour when added. Interestingly, the increase in the cactus gum concentration from 1% to 3% had no effect on the wheat flour’s PT. Wheat flour and corn starch both had a lower PT after being treated with gum Cordia at a 2% concentration as opposed to a 1% concentration [32,40].

### 3.3. Textural Properties of Flour-Gum Mixtures

The paste of the wheat flour blends with acacia or cactus gum gels upon resting and the texture of the gels was estimated by TPA. Gel texture is greatly affected by the presence of additives such as gums and stabilizers, which are used as processing aids in the food industry. The hardness of the flour gels is an important parameter to estimate their ease of mastication and swallowing. The gel hardness was found to be highest with the addition of cactus gum at 1%, which was higher than the control wheat flour gel (Table 3). A lower CG might have induced phase separation, enhanced the amylose–amylose interaction, and increased the retrogradation [41]. The increased hardness of the gel might also result from the immobilization of water molecules by the gum and increase the effective concentration of starch in the system. However, the presence of 3% cactus gum provided a softer gel than the control, with a hardness value of 57 N. Nonetheless, the least hardness, or the softest and most pliable textural hardness, was noticed with the blending of acacia gum at 3%, where a difference of 22 N was observed compared to the highest hardness of 63.3 N for wheat flour gels with 1% cactus gum. This reduction in the hardness could be due to the possible formation of hydrogen bonds between the gum and the amylose, which interferes with the formation of an organized structure during starch recrystallization. Thus, compared to the cactus, the acacia gums were better at controlling the hardness of the wheat flour gels, which, in turn, controlled the recrystallization of wheat starch. Our findings also agree with those of Alamri et al. [32], who found that the hardness of wheat flour gels was lowered by the addition of ziziphus gum.

Gumminess is described as the amount of energy required to disintegrate the gel before swallowing and is a product of hardness and cohesiveness. The highest gumminess of 31 N was noticed for the wheat flour with 1% cactus gum, although this was statistically similar to that of the control wheat flour gel. Interestingly, lower concentrations (1%) of both gums (acacia and cactus gum) did not lead to much variation in the gumminess of gels. However, at higher levels (3%), a significant reduction in gumminess was noticed, where the lowest gumminess was observed by the acacia gum blends with wheat flour. Thus, it could be estimated that for a richer gummy texture, lower levels of gum would perform better than higher levels. Ziziphus gum made the wheat flour stickier, which backs up the current findings [32].

The chewiness is the amount of energy required to masticate the gel before swallowing. The control gel exhibited the highest chewiness and remained somewhat similar to the gel with 1% cactus gum. The chewiness of the gels was reduced with gums, especially when added at higher concentrations, and the least-chewy gel was obtained in the presence of 3% acacia gum in the blend. The addition of acacia gum presented a 27% reduction in the chewiness of the wheat flour gels. As Alamri et al. [32] showed, the addition of 2% gum Cordia made the wheat flour gels less chewy than the control gels.

Springiness is the regained shape of the gel between the end of the first bite and the start of the second one. The wheat flour gels provided the highest springiness, with a value of 10 mm, indicating the chewiest nature. Although the addition of gums led to a significant (*p* < 0.05) reduction in the springiness of the gels, this shows that the addition of gums resulted in a slower formation of polymer aggregates and provided more viscous regions and less springy gels. However, interestingly, both the gums showed a comparable springiness at a higher level of addition (3%). Nonetheless, the least springy gel was observed when cactus gum was added at 1%. Thus, this shows that the presence of acacia gum at a lower level would provide springy gels. Alamri et al. [32] also found that when crude ziziphus gum was added to wheat flour, the gel’s springiness decreased according to the amount that was added.

The energy that is needed to overcome the internal bond strength within the gel body is termed “cohesiveness” and varies with the additions, additives, and concentrations. The wheat flour gels with acacia gum presented the highest cohesiveness. This shows that the gel is more resistant to breakage with the addition of cactus gum. On the other hand, the least-cohesive gel body was noticed when cactus gum was added at a level of 3%. A more cohesive nature is preferable as the gel should not be brittle upon chewing and must not shatter under force. Thus, acacia gum could be a better choice if a cohesive gel body is desired. Adding gum Cordia to wheat flour [32] led to a rise in the strength of the dough.

Adhesiveness is the sum of the attractive forces prevailing at the boundary between the food surface and the container walls. It also refers to the work required to pull the food away from the surface of the container. The wheat flour gels with 1% acacia provided a somewhat similar adhesiveness to the control. However, the least-adhesive nature was observed for the gels with 1% cactus gum in the blend, where a 29% reduction in the adhesiveness was observed compared to the control wheat flour. The gels with 3% gum acacia gum displayed the same adhesiveness as the gels with 3% acacia. Thus, to obtain an adhesive gel, the addition of acacia could be recommended at 1%. Lower adhesiveness could be explained by the lower starch–starch interaction due to the presence of gums. Also, when the gum Cordia was added to the wheat flour gels, it made them less cohesive [32].

### 3.4. Crumb-Color Profiles of Baked Products

Color is an extremely essential factor in attracting customers and inducing buying intent [29]. The presence of substances in the formula has a big impact on how the bread crumb color develops (Table 4). The lightest (L*) of the bread crumb was visible after the cactus gum was added. When acacia gum was added, however, the whiteness of the crumb was diminished (72.7) compared to the control (75.3). The increased browning generated by the inclusion of acacia gum could be the source of the bread’s diminished lightness. Similarly, as with the bread tests, the sample treated with cactus gum had the highest lightness. The addition of acacia gum, on the other hand, lowered the lightness of the cake crumb, with the cake crumb at three percent having the lowest level of lightness (69.6). Increasing the amount of gum Cordia and ziziphus gum in bread decreased the lightness of the bread and cakes [32].

In terms of greenness (−a*), the bread with a percentage of cactus gum added had the highest values. The inclusion of acacia, on the other hand, decreased the greenness of the bread crumb by a factor of −6.48. Overall, increasing the gum concentration from 1% to 3% considerably changed the greenness of the crumb, although acacia and cactus behaved in opposing ways, with cactus lowering the greenness and acacia achieving the opposite. When a larger level of acacia gum was applied to the cake crumbs, the crumbs turned greener (3%). The cake with the cactus gum, on the other hand, had the fewest green crumbs, and the color of the crumbs remained constant regardless of the cactus gum concentration. When gum Cordia was added to bread and cake, it was found to reduce the greenness, especially at higher concentrations [32].

The yellowness (b*) of the crumb, on the other hand, was higher in the presence of gums, especially at higher concentrations (3%). The bread with the highest yellowness (20.65) was made with the addition of 10% cactus gum. Despite this, the yellowness of the crumbs did not significantly alter as the acacia concentration increased. The control bread, which did not include the gums, had the lowest amount of yellowness (17.31), as expected. These reduced yellowness readings in the control bread indicate that the gums have interacted with the gluten proteins and caused color changes in the final baked bread. The presence of acacia gum at 3% produced the highest yellowness (29.4) in the crumb in the case of the cakes, followed by the control sample without any gum. The cake crumbs had the least yellowness (25.2) when cactus gum was added. When egg proteins were added to the cake crumbs, they may have interacted with the acacia gum, resulting in more yellowness than the control. The addition of guar and xanthan gums, on the other hand, boosted the yellowness of the purple yam muffin [42]. According to Alamri, Mohamed, Hussain, Ibraheem, Qasem, Shamlan, Hakeem, and Ababtain [32], adding gum corrida made bread less yellow, especially at higher levels.

### 3.5. Volume, Weight, and Specific Volume of Baked Products

**Figure 2:**
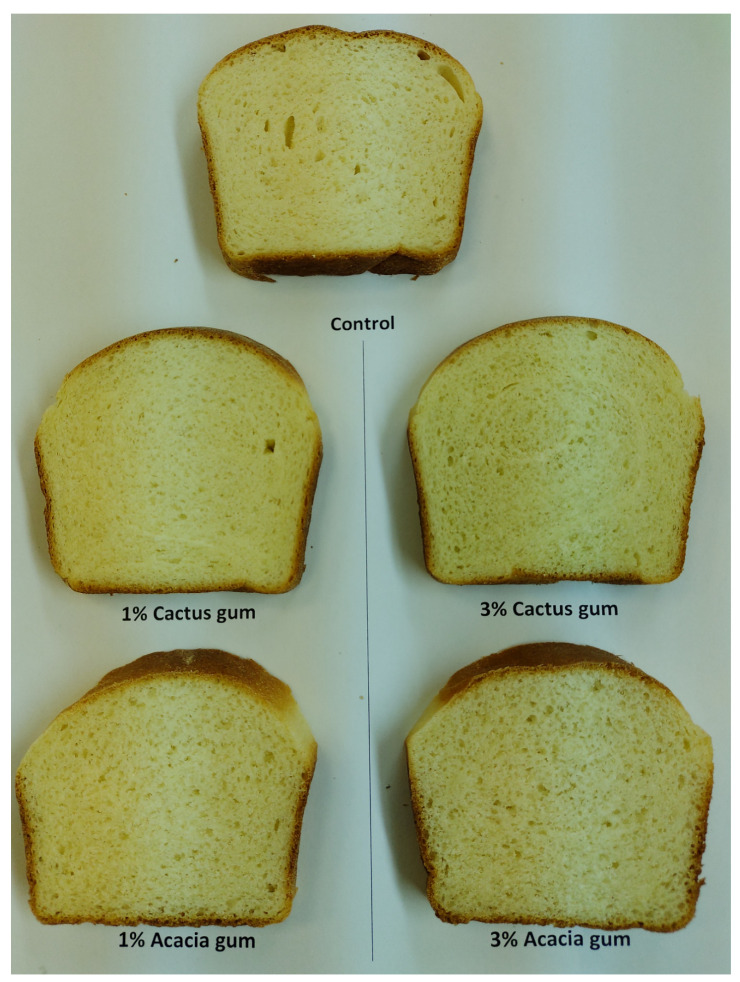
Images of the central slices of the different breads containing acacia and cactus gums.

**Figure 3:**
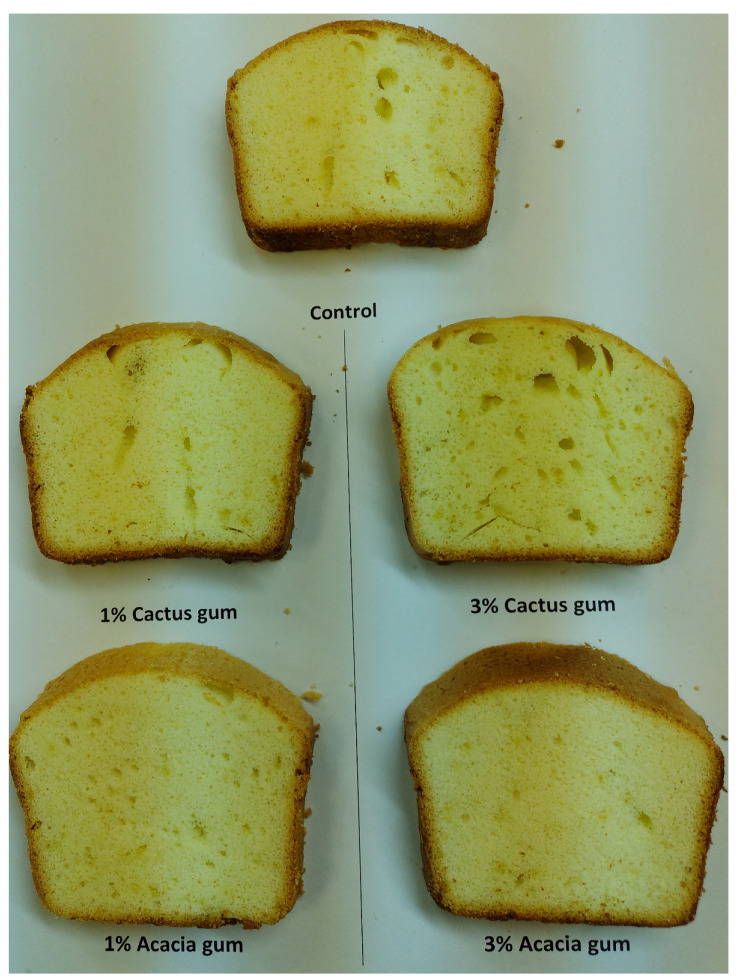
Images of the cake loaves containing acacia and cactus gums.

Aside from color and shape, the volume of baked goods is one of the most enticing features to draw customers’ attention. The entrapment of CO_2_ created during fermentation, which is held in the form of uniform microscopic air bubbles in the crumb structure, gives rise to the volume of leavened bread products. The volume of the bread crumb is influenced more by the regularity of bubble size and shape [43]. It has been proposed that the type of gum and the concentration of the gum applied to the cooked loaf have a significant impact on the increase in loaf volume [44]. The bread with acacia gum had the largest volume of 832 cm^3^ (Table 5). The crumb digital photos (Figure 2) confirmed the volume data, with the bread having 3% cactus and acacia gums, indicating the most porous and aerated structure. The inclusion of cactus gum, on the other hand, resulted in a lower rise in the bread loaf. In comparison with the loaf with 1% gums, a higher concentration (3%) of both gums resulted in a higher loaf volume. This suggests that increasing the gum content increased the gas-holding capacity of the gas cells. The hydrated chains of gums lose water when heated during baking and the water of hydration is released, permitting the creation of stronger interchain temporal networks in bread crumbs. These networks aid in the retention of gas in the dough during the early stages of baking, reducing gas loss and increasing bread volume. The most compact bread, on the other hand, was made without the addition of gums, being made only with wheat flour. In the case of the sponge cake, the components have a direct impact on the volume of the cake, particularly those that control the aeration and foam stability of the batter. The sponge cake volume ranged from 811 to 709 cm^3^. In comparison with the control, the addition of acacia gums (1 and 3%) made the cake bigger. The addition of cactus gum, on the other hand, resulted in a reduction in cake volume, with the most compact cake being the one with 3%, which had the smallest volume of 709 cm^3^. The impact of the gum could be attributed to its viscosity, which enhanced batter thickness and regulated air diffusion, resulting in a volume increase early in the baking process [32]. Furthermore, the gelatinization temperature of the wheat in the presence of gums affects the viscosity of the batter. The greater the potential viscosity, the higher the gelatinization temperature, which favors cake volume [45]. A rise in the gelatinization temperature of starches treated with gum Cordia and ziziphus gum was previously documented [46]. As a result, we observed an increase in cake volume with the addition of acacia gum in this investigation. The digital image of the cake (Figure 3) revealed a more regular and frequent cell density in the crumb, which could explain the increase in cake volume. The cactus, on the other hand, gave the cake crumbs uneven, long air cells and less air.

Regarding the weight of the bread loaves, the control bread with the smallest volume had the highest weight (326 g). The bread with 1% acacia was found to have the lowest weight (317 g). The presence of a higher gum content, on the other hand, resulted in better water retention and less weight loss than the bread with 1% gum. Overall, there was little difference in the weight of the loaves. This means that the presence of hydrophilic gums was unable to bind to a significant amount of water at a high enough baking temperature, resulting in a similar weight to the control. The cake made with 3% cactus gum, on the other hand, had the highest weight (321 g). With a weight of 286 g, the cake without gum was the lightest.

The specific volume is the quotient of the volume and weight of leavened baked goods. When comparing breads with the higher gum concentrations (3%) of acacia and cactus to loaves with 1% gum, it was discovered that the breads with the higher gum concentrations (3%) had higher specific volumes. As a result of its maximum weight, the control bread had the lowest specific volume (2.37). The control cake had the highest specific volume, with a value of 2.62, due to its thicker crumbs and higher weight. When pullulan, xanthan gum, and pectin were added to the bread, Al-Dalain and Morsy [34] noticed an increase in volume and specific volume.

### 3.6. Textural Attributes of Baked Products

The quality of bakery items degrades over time due to starch retrogradation, which causes the amylose to recrystallize, resulting in increased hardness. One of the key elements in improving the stiffness of the bread is the moisture migration from the crumbs to the surface [33]. As a result, the bread and cake quality were evaluated after 24 and 96 h of storage. The control bread had the highest stiffness (493 g) after 24 h according to the texture analyzer (Table 6). When cactus gum was combined with wheat flour, however, the bread was the least firm. After 24 h of storage, all the bread samples with gum, whether cactus or acacia, had a lower hardness, indicating that the gums soften the crumb. Bread-crumb hardness is generally lower in samples with increased moisture content. As a result of the higher moisture percentage in the crumbs after 24 h of storage, less firmness might be predicted. However, because the trend in the water absorption of the control was the highest, and the firmest texture was observed for the same sample, the softness of the bread with gums could not be exclusively connected to the dough–water absorption during dough mixing, as observed by DoughLab. After 96 h of storage, the control bread showed an almost twofold increase in firmness and was the firmest, supporting the control’s lack of moisture-preserving abilities due to the lack of gums. In contrast, after 96 h, the bread with 1% cactus gum had a one-third magnitude of hardness compared to the control. This shows that gums affect bread stiffness by restricting amylose–amylose interactions and favoring amylose–protein or gum–amylose interactions [47]. As can be seen from the firmness data, the inclusion of gums kept the firmness of the breads consistently lower than the control across the storage periods, resulting in considerable crumb softening. After 24 or 96 h, however, the springiness could not be determined.

In the case of the cakes, the firmness of the cakes after 24 and 96 h of storage followed the same pattern. The cake crumb with 3% cactus gum had the firmest crumb texture after 24 h, with a firmness value of 252 g, followed by the control crumb (234 g). The cake with 3% acacia, on the other hand, stayed softer than the other cake crumbs. In keeping with the firmness trend observed for crumbs after 24 h, the samples showed a similar tendency of reduced firmness after 96 h of storage. The control cake with no added gum had the highest firmness (438 g), but the cake with 3% acacia had the least firmness after 96 h. The varying degrees of interaction between the starch and different types of gums, which modify the retrogradation behavior of the cake crumb, could explain the variance in cake firmness. When xanthan gum was used instead of HMC, the cake was noticeably firmer [13]. Surprisingly, there was no obvious link between the moisture content of the cake and its stiffness. According to the springiness statistics, the cake with cactus gum (3%) had the highest springiness values after 24 h. The cake with the same concentration of cactus gum remained springier in texture when the storage duration (96 h) was increased. In contrast to cactus gum, the cake with the greatest added amount of acacia (3%) had the least-springy cake crumb after 24 h, but after 96 h the trend shifted and the cake with the most cactus gum had the least springiness, with a value of 47.6. Overall, the springiness of the cake was not affected as much as its hardness when the storage time was changed.

### 3.7. Sensory Quality of Baked Products

**Figure 4:**
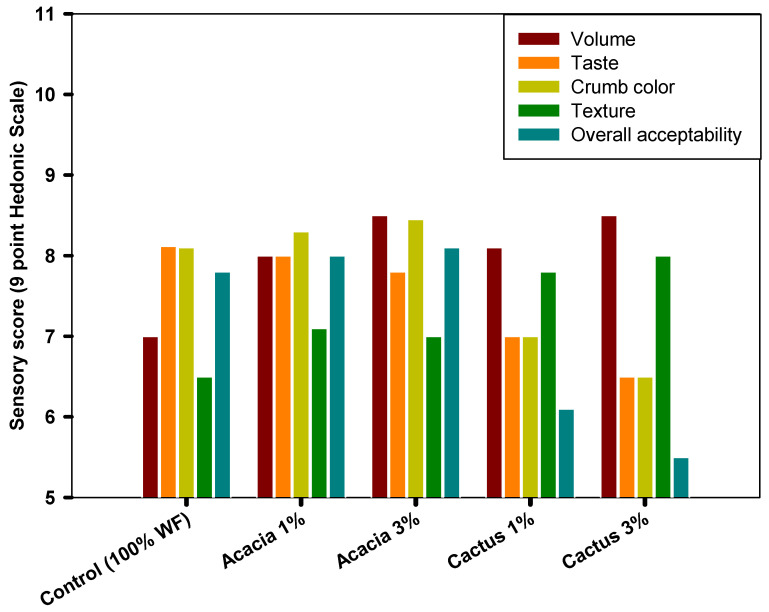
Sensory score of bread containing acacia and cactus gums.

The sensory qualities of leavened baked goods are primarily determined by the volume, color, flavor, texture, and overall acceptability of the end product to replicate consumer preferences [32]. The means of the hedonic scores for the sensory measures listed above are shown in Figure 4. In terms of volume, the bread made with various blends of wheat flour and gums came in first place. The bread made with 3% cactus gum and acacia gum came in second place. This preference for bread with a higher gum concentration may be explained by the crumbs’ thin-walled yet extended air cells (Figure 1). The participants’ least preferred samples remained the bread with lower gum concentrations and the control bread. The sensory data on bread volume preference agreed with the volume data, which showed that when gums were added at 3%, the volume rose.

Similarly, the panelists preferred the bread loaf with 3% acacia for its crumb color, followed by the bread with 1% acacia. However, the bread with the lowest ranking was baked with 3% cactus gum. This implies that the presence of cactus gum was not desired in the bread due to the possibility of a change in the bread’s lighter yellow color to a more greenish hue, which was deemed undesirable. On the other hand, the best-tasting bread was the control with no added gums, followed by the acacia-containing breads. Panelists did not like the addition of cactus gum to the bread at a concentration of 1 or 3%. This led to lower hedonic scores for the bread samples that included this gum.

The bread loaves with cactus gum (1 or 3%) were more accepted for their texture than those containing acacia gum. This demonstrates that the inclusion of gums improved the bread’s texture acceptability. Despite this, the control bread received the lowest rating of all the samples. This low acceptability of the control could be a result of the crumb having the highest instrumental stiffness in comparison to the other samples. In general, the overall acceptability is the sum of all the hedonic scores obtained for the various factors, and it corresponds to the amount at which a loaf is likely to be accepted [46]. The bread with 3% acacia gum had the highest overall approval and the highest rate for preference and choice. On the other hand, the bread with 3% cactus gum received the lowest overall acceptance score. This suggests that the taste and appearance of the loaves had a significant impact on how the panelists rated the bread’s overall acceptability. Alamri, Mohamed, Hussain, Ibraheem, Qasem, Shamlan, Hakeem, and Ababtain [32] found that adding 2% ziziphus gum to bread made it more popular.

**Figure 5:**
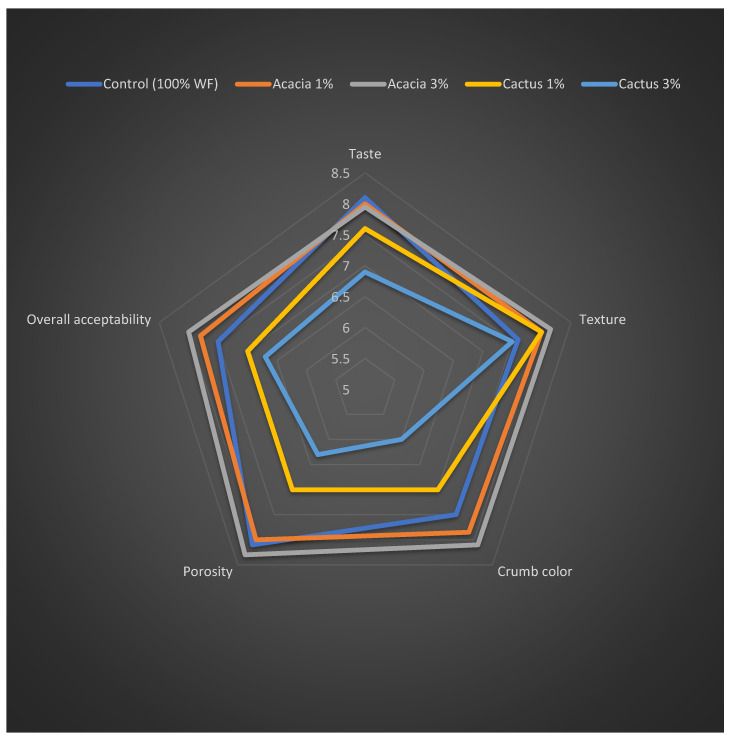
Sensory scores for cakes containing acacia and cactus gums.

The pictures of the cake samples baked with or without the presence of gums are presented in Figure 3, whereas the means of the hedonic scores for the sponge cakes are displayed in a spiderweb graph (Figure 5). In terms of flavor, the control sample was the most preferred, and the presence of gums resulted in the counterparts receiving a lower ranking. Similar to the bread, the cake with cactus gum remained unpopular, particularly in higher quantities (3%). By and large, the presence of gums in food systems enables a more gradual release of flavoring chemicals during mastication [48]. However, after a certain level of gum is reached, the normal flavor of the cake becomes bland or less rich, which has a detrimental effect on consumer preference [13]. As with the bread sample, the cake’s crumb color was preferred when 3% acacia gum was added. On the other hand, adding 3% cactus gum produced the least-acceptable color. Similarly, the cake containing 3% acacia received the highest rating for color choice, followed by the control cake. Nonetheless, the addition of cactus gum, particularly at a concentration of 3%, reduced the cake’s texture acceptability, earning it only 7.5 out of a possible 9 points. Prior to this, we found that adding 2% gum Cordia to cakes made them look and taste bad [33].

Porosity is a critical feature in determining the sponge cake’s sponginess and fluffy texture. The addition of 3% acacia gum resulted in a crumb that was even more porous than the control, hence, this remained the best sample. Figure 4 depicts the cake’s uniform but homogeneous porous crumb cells, which contain 3% more acacia than the other samples. In comparison with the control and the other gum-containing samples, the inclusion of cactus gum resulted in the relatively larger non-homogenous pores of the crumb, which could have resulted in the lowest hedonic scores and the lowest level of acceptance. Thus, the cake’s overall acceptability remained higher when acacia gum was added in comparison with the control, as well as when cactus gum was added. The inclusion of xanthan gum decreased the sponge’s overall acceptability [13].

## 4. Conclusions

Acacia gum significantly (*p* ≤ 0.05) improved the dough’s stability compared to the control and the sample with cactus gum. The gum addition had a detrimental effect on the pasting characteristics of the flour dispersions. The gums imparted a softer gel texture to wheat flour, particularly at a higher level of addition (3%). Although cactus and acacia gums have a marked difference in their ability to alter the color of bread and cake, gum acacia increased the volume of the bread and sponge cake. The addition of cactus gum decreased the bread’s hardness and inhibited staling after 24 and 96 h of storage, but the addition of acacia gum resulted in a softer cake under the same conditions. Additionally, the inclusion of acacia gum, particularly at a concentration of 3%, increased sensory properties and overall acceptance. As a result, adding acacia gum to pan bread and sponge cake results in a superior-quality product with a softer texture.

## Figures and Tables

**Table 1:** Effect of acacia and cactus gums on the dough mixing properties.

	WA (%)	DDT (min)	Stability (min)	Softening (FU)	MTI (FU)	Quality Number
Control (100% WF)	61.47 ± 1.16 ^a^	1.60 ± 0.08 ^c^	5.70 ± 0.22 ^c^	91.67 ± 2.36 ^e^	35.67 ± 4.19 ^e^	61.23 ± 0.95 ^a^
Acacia 1%	59.93 ± 0.09 ^b^	5.50 ± 0.08 ^a^	6.53 ± 0.12 ^b^	105.67 ± 0.94 ^c^	64.67 ± 0.47 ^c^	53.00 ± 0.73 ^c^
Acacia 3%	58.63 ± 0.12 ^d^	5.57 ± 0.26 ^a^	7.40 ± 0.16 ^a^	99.67 ± 0.47 ^d^	52.33 ± 2.05 ^d^	56.43 ± 0.39 ^b^
Cactus 1%	59.50 ± 0.41 ^bc^	3.90 ± 0.08 ^b^	4.20 ± 0.16 ^d^	131.93 ± 2.15 ^b^	100.67 ± 4.19 ^b^	42.99 ± 0.74 ^d^
Cactus 3%	58.93 ± 0.09 ^c^	3.93 ± 0.09 ^b^	3.40 ± 0.08 ^e^	146.63 ± 1.73 ^a^	111.00 ± 2.94 ^a^	40.97 ± 1.19 ^e^

WF = wheat flour; WA = water absorption; DDT = dough development time; MTI = mixing tolerance index; FU = Farinograph units. Values followed by different letters in columns are significantly different at *p* < 0.05.

**Table 2:** Effect of acacia and cactus gums on the pasting properties of flours.

	PV (cP)	BD (cP)	FV (cP)	SB (cP)	PT (°C)
Control (100% WF)	1886 ± 35.26 ^a^	736 ± 21.79 ^a^	2056 ± 1.70 ^a^	905 ± 12.36 ^a^	69.40 ± 0.04 ^c^
Acacia 1%	1693 ± 4.97 ^b^	647 ± 9.46 ^c^	1860 ± 1.25 ^c^	815 ± 5.35 ^d^	83.68 ± 0.31 ^b^
Acacia 3%	1453 ± 6.55 ^c^	568 ± 9.46 ^d^	1593 ± 25.77 ^d^	705 ± 9.90 ^e^	84.07 ± 0.65 ^a^
Cactus 1%	1854 ± 7.04 ^a^	732 ± 4.85 ^a^	2004 ± 9.84 ^b^	881 ± 8.99 ^b^	68.53 ± 0.02 ^d^
Cactus 3%	1847 ± 11.02 ^a^	702 ± 0.70 ^b^	1991 ± 13.10 ^b^	848 ± 0.47 ^c^	68.54 ± 0.01 ^d^

WF = wheat flour; PV = peak viscosity; BD = breakdown viscosity; FV = final viscosity; SB = setback viscosity; PT = pasting temperature; cP = centipoise; °C = centigrade. Values followed by different letters in columns are significantly different at *p* < 0.05.

**Table 3:** Effect of acacia and cactus gums on the TPA properties of flour gels.

	Hardness (N)	Gumminess (N)	Chewiness (N.mm)	Springiness (mm)	Cohesiveness	Adhesiveness (mJ)
Control (100% WF)	61.00 ± 0.65 ^b^	30.00 ± 0.38 ^ab^	297 ± 1.88 ^a^	10.00 ± 0.07 ^a^	0.49 ± 0.01 ^b^	0.83 ± 0.05 ^a^
Acacia 1%	55.10 ± 0.81 ^c^	28.59 ± 0.47 ^b^	284 ± 3.55 ^b^	9.95 ± 0.04 ^a^	0.52 ± 0.02 ^a^	0.87 ± 0.05 ^a^
Acacia 3%	41.33 ± 0.47 ^d^	22.04 ± 0.23 ^d^	216 ± 2.22 ^c^	9.80 ± 0.01 ^b^	0.53 ± 0.01 ^a^	0.70 ± 0.02 ^b^
Cactus 1%	63.33 ± 0.45 ^a^	31.04 ± 0.73 ^a^	303 ± 5.85 ^a^	9.75 ± 0.04 ^b^	0.49 ± 0.02 ^b^	0.60 ± 0.06 ^c^
Cactus 3%	57.00 ± 0.72 ^c^	26.78 ± 0.08 ^c^	262 ± 0.80 ^c^	9.80 ± 0.02 ^b^	0.47 ± 0.01 ^c^	0.70 ± 0.01 ^b^

WF = wheat flour; N = Newton; N.mm = Newton millimeter; mm = millimeter; mJ = milli Joul. Values followed by different letters in columns are significantly different at *p* < 0.05.

**Table 4:** Effect of acacia and cactus gums on the crumb-color parameters of bread and cake samples.

	L*	a*	b*
	Pan bread		
Control (100% WF)	75.39 ± 0.28 ^b^	−7.05 ± 0.02 ^c^	17.31 ± 0.02 ^c^
Acacia 1%	74.54 ± 0.16 ^b^	−6.89 ± 0.01 ^d^	17.58 ± 0.07 ^c^
Acacia 3%	72.75 ± 0.28 ^c^	−6.48 ± 0.06 ^e^	17.97 ± 0.10 ^c^
Cactus 1%	78.27 ± 0.48 ^a^	−7.33 ± 0.05 ^b^	18.75 ± 0.12 ^b^
Cactus 3%	78.77 ± 0.04 ^a^	−7.68 ± 0.01 ^a^	20.65 ± 0.02 ^a^
	Sponge cake		
Control (100% WF)	71.53 ± 0.04 ^c^	−9.28 ± 0.01 ^c^	26.66 ± 0.03 ^b^
Acacia 1%	71.21 ± 0.08 ^d^	−9.39 ± 0.07 ^b^	26.12 ± 0.08 ^b^
Acacia 3%	69.63 ± 0.01 ^e^	−9.65 ± 0.05 ^a^	29.48 ± 0.18 ^a^
Cactus 1%	71.94 ± 0.04 ^b^	−9.15 ± 0.07 ^d^	25.40 ± 0.06 ^c^
Cactus 3%	73.45 ± 0.04 ^a^	−9.21 ± 0.02 ^d^	25.23 ± 0.07 ^c^

WF = wheat flour; L* = lightness; a* = green/magenta; b* = blue/yellow. Values followed by different letters in columns (under bread or cake) are significantly different at *p* < 0.05.

**Table 5:** Effect of acacia and cactus gums on the volume, weight, and specific volume of bread and cake samples.

	Loaf Volume (cm^3^)	Loaf Weight (g)	Specific Volume (cm^3^/g)
	Pan bread		
Control (100% WF)	771 ± 5.35 ^e^	326 ± 3.30 ^a^	2.37 ± 0.02 ^c^
Cactus 1%	801 ± 3.30 ^c^	317 ± 1.25 ^c^	2.52 ± 0.02 ^b^
Cactus 3%	821 ± 4.19 ^b^	320 ± 1.70 ^bc^	2.57 ± 0.01 ^a^
Acacia 1%	790 ± 4.50 ^d^	318 ± 1.63 ^c^	2.49 ± 0.03 ^b^
Acacia 3%	832 ± 2.05 ^a^	321 ± 2.49 ^ab^	2.59 ± 0.02 ^a^
	Sponge cake		
Control (100% WF)	749 ± 2.94 ^c^	286 ± 1.25 ^c^	2.62 ± 0.02 ^a^
Cactus 1%	732 ± 4.97 ^d^	289 ± 0.82 ^c^	2.53 ± 0.04 ^bc^
Cactus 3%	709 ± 3.30 ^e^	321 ± 3.74 ^a^	2.21 ± 0.03 ^e^
Acacia 1%	785 ± 4.08 ^b^	318 ± 2.05 ^ab^	2.47 ± 0.03 ^d^
Acacia 3%	811 ± 3.30 ^a^	315 ± 1.63 ^b^	2.57 ± 0.02 ^b^

WF = wheat flour; Values followed by different letters in columns (under bread or cake) are significantly different at *p* < 0.05.

**Table 6:** Effect of acacia and cactus gums on the textural profile of bread and cake samples.

	Firmness (g) 24 h	Springiness (%) 24 h	Firmness (g) 96 h	Springiness (%) 96 h
	Pan bread			
Control (100% WF)	493.00 ± 16.38 ^a^	-	1070.57 ± 30.54 ^a^	-
Cactus 1%	232.58 ± 17.65 ^d^	-	357.88 ± 35.08 ^d^	-
Cactus 3%	256.25 ± 17.65 ^cd^	-	473.81 ± 24.23 ^bc^	-
Acacia 1%	282.03 ± 17.88 ^bc^	-	497.25 ± 15.90 ^b^	-
Acacia 3%	303.84 ± 6.16 ^b^	-	500.40 ± 35.40 ^b^	-
	Sponge cake			
Control (100% WF)	234.29 ± 15.86 ^ab^	52.29 ± 1.18 ^b^	438.92 ± 30.74 ^a^	47.95 ± 0.86 b
Cactus 1%	174.37 ± 5.91 ^c^	55.22 ± 0.47 ^a^	318.26 ± 8.65 ^c^	47.61 ± 1.01 ^b^
Cactus 3%	252.26 ± 27.76 ^a^	54.45 ± 0.41 ^a^	356.37 ± 8.21 ^b^	51.34 ± 0.93 ^a^
Acacia 1%	205.74 ± 15.17 ^b^	53.36 ± 0.10 ^ab^	305.20 ± 5.99 ^cd^	51.38 ± 0.18 ^a^
Acacia 3%	161.75 ± 10.32 ^c^	52.15 ± 1.65 ^b^	215.67 ± 5.42 ^e^	48.31 ± 0.56 ^b^

WF = wheat flour; Values followed by different letters in columns (under bread or cake) are significantly different at *p* < 0.05.

## Data Availability

No new data were created or analyzed in this study. Data sharing is not applicable to this article.

## References

[1] Awulachew M. (2021). Gluten-Free Bread: A Review of Non-Gluten Component Characteristics Retain the Baking Quality and Viscoelastic Properties of Dough. Gl J Foo Sci Nutri GJFSN.

[2] Gänzle M., Gobbetti M. (2012). 12.1 Microbial Ecology of Sourdough. Handbook on Sourdough Biotechnology.

[3] Itthivadhanapong P., Jantathai S., Schleining G. (2016). Improvement of physical properties of gluten-free steamed cake based on black waxy rice flour using different hydrocolloids. J. Food Sci. Technol..

[4] Hojjatoleslami M., Azizi M.H. (2015). Impact of tragacanth and xanthan gums on the physical and textural characteristics of gluten-free cake. Nutr. Food Sci. Res..

[5] Jia C., Huang W., Ji L., Zhang L., Li N., Li Y. (2014). Improvement of hydrocolloid characteristics added to angel food cake by modifying the thermal and physical properties of frozen batter. Food Hydrocoll..

[6] Rodge A., Sonkamble S., Salve R., Hashmi S. (2012). Effect of hydrocolloid (guar gum) incorporation on the quality characteristics of bread. J. Food Processing Technol..

[7] Kohajdová Z., Karovičová J. (2009). Application of hydrocolloids as baking improvers. Chem. Pap..

[8] Ma M., Mu T., Sun H., Zhou L. (2022). Evaluation of texture, retrogradation enthalpy, water mobility, and anti-staling effects of enzymes and hydrocolloids in potato steamed bread. Food Chem..

[9] Sharadanant R., Khan K. (2006). Effect of hydrophilic gums on the quality of frozen dough: Electron microscopy, protein solubility, and electrophoresis studies. Cereal Chem..

[10] Aboulnaga E., Ibrahim F.Y., Youssif M., Mohamed A. (2018). Influence of various hydrocolloids addition on pan bread quality. J. Food Dairy Sci..

[11] Barcenas M.E., Rosell C.M. (2005). Effect of HPMC addition on the microstructure, quality and aging of wheat bread. Food Hydrocoll..

[12] Nessrien M., Gadallah M. (2011). Effect of adding different gums and emulsifiers on quality attributes and staling rate of microwave-baked cakes. Alex. J. Food Sci. Technol..

[13] Noorlaila A., Hasanah H.N., Asmeda R., Yusoff A. (2020). The effects of xanthan gum and hydroxypropylmethylcellulose on physical properties of sponge cakes. J. Saudi Soc. Agric. Sci..

[14] Salehi F. (2020). Effect of common and new gums on the quality, physical, and textural properties of bakery products: A review. J. Texture Stud..

[15] Salehi F., Kashaninejad M. (2018). Modeling of xanthan gum effect on textural properties of carrot cake. Iran. J. Food Sci. Technol..

[16] Turabi E., Sumnu G., Sahin S. (2010). Quantitative analysis of macro and micro-structure of gluten-free rice cakes containing different types of gums baked in different ovens. Food Hydrocoll..

[17] Feugang J.M., Konarski P., Zou D., Stintzing F.C., Zou C. (2006). Nutritional and medicinal use of Cactus pear (*Opuntia* spp.) cladodes and fruits. Front. Biosci..

[18] Sepúlveda E., Sáenz C., Aliaga E., Aceituno C. (2007). Extraction and characterization of mucilage in *Opuntia* spp.. J. Arid Environ..

[19] Stintzing F.C., Carle R. (2005). Cactus stems (*Opuntia* spp.): A review on their chemistry, technology, and uses. Mol. Nutr. Food Res..

[20] Salehi E., Emam-Djomeh Z., Askari G., Fathi M. (2019). Opuntia ficus indica fruit gum: Extraction, characterization, antioxidant activity and functional properties. Carbohydr. Polym..

[21] Dick M., Dal Magro L., Rodrigues R.C., de Oliveira Rios A., Flôres S.H. (2019). Valorization of Opuntia monacantha (Willd.) Haw. cladodes to obtain a mucilage with hydrocolloid features: Physicochemical and functional performance. Int. J. Biol. Macromol..

[22] Manhivi V.E., Venter S., Amonsou E.O., Kudanga T. (2018). Composition, thermal and rheological properties of polysaccharides from amadumbe (*Colocasia esculenta*) and cactus (*Opuntia* spp.). Carbohydr. Polym..

[23] De Waal H., Louhaichi M., Taguchi M., Fouché H., De Wit M. Development of a cactus pear agro-industry for the sub-Sahara Africa Region. Proceedings of the International Workshop, University of the Free State.

[24] Hussain S., Mohamed A.A., Alamri M.S., Ibraheem M.A., Qasem A.A.A., Alsulami T., Ababtain I.A. (2022). Effect of Cactus (*Opuntia ficus-indica*) and Acacia (*Acacia seyal*) Gums on the Pasting, Thermal, Textural, and Rheological Properties of Corn, Sweet Potato, and Turkish Bean Starches. Molecules.

[25] Alamri M.S., Mohamed A., Hussain S., Al-Ruquie I. (2014). Behri dates pits-enriched bread: Effect on dough rheology, bread quality, and shelf life. Ital. J. Food Sci..

[26] Dangi P., Chaudhary N., Khatkar B. (2019). Rheological and microstructural characteristics of low molecular weight glutenin subunits of commercial wheats. Food Chem..

[27] AACC (2000). Approved methods of the American association of cereal chemists. Amer Assn of Cereal Chemists.

[28] Alamri M.S. (2014). Okra-gum fortified bread: Formulation and quality. J. Food Sci. Technol..

[29] Mahmood K., Alamri M., Mohamed A., Hussain S., Abdu Qasem A. (2015). Gum cordia: Physico-functional properties and effect on dough rheology and pan bread quality. Qual. Assur. Saf. Crops Foods.

[30] Wang J.J., Liu G., Huang Y.-B., Zeng Q.-H., Song G.-S., Hou Y., Li L., Hu S.-Q. (2016). Role of N-terminal domain of HMW 1Dx5 in the functional and structural properties of wheat dough. Food Chem..

[31] Chen M., Wang L., Qian H., Zhang H., Li Y., Wu G., Qi X. (2019). The effects of phosphate salts on the pasting, mixing and noodle-making performance of wheat flour. Food Chem..

[32] Alamri M.S., Mohamed A.A., Hussain S., Ibraheem M.A., Qasem A.A.A., Shamlan G., Hakeem M.J., Ababtain I.A. (2022). Functionality of Cordia and Ziziphus Gums with Respect to the Dough Properties and Baking Performance of Stored Pan Bread and Sponge Cakes. Foods.

[33] Huang Z., Wang J.J., Chen Y., Wei N., Hou Y., Bai W., Hu S.-Q. (2020). Effect of water-soluble dietary fiber resistant dextrin on flour and bread qualities. Food Chem..

[34] Al-Dalain S.Y., Morsy M.K. (2018). Effect of Pullulan and Hydrocolloids on Rheological Properties and Quality Parameters of Wheat-Soy Baladi Bread. Food Nutr. Sci..

[35] Mahmood K., Alamri M.S., Abdellatif M.A., Hussain S., Qasem A.A.A. (2018). Wheat flour and gum cordia composite system: Pasting, rheology and texture studies. Food Sci. Technol..

[36] Singh A., Geveke D.J., Yadav M.P. (2017). Improvement of rheological, thermal and functional properties of tapioca starch by using gum arabic. LWT.

[37] Shrivastava M., Yadav R.B., Yadav B.S., Dangi N. (2018). Effect of incorporation of hydrocolloids on the physicochemical, pasting and rheological properties of colocasia starch. J. Food Meas. Charact..

[38] Alamri M.S., Mohamed A.A., Hussain S. (2013). Effects of alkaline-soluble okra gum on rheological and thermal properties of systems with wheat or corn starch. Food Hydrocoll..

[39] Kiprop V.J., Omwamba M.N., Mahungu S.M. (2021). Influence of Gum Arabic from Acacia Senegal var. kerensis on the Modifications of Pasting and Textural Properties of Cassava and Corn Starches. Food Nutr. Sci..

[40] Hussain S., Mohamed A.A., Alamri M.S., Ibraheem M.A., Qasem A.A.A., Shahzad S.A., Ababtain I.A. (2020). Use of Gum Cordia (*Cordia myxa*) as a Natural Starch Modifier; Effect on Pasting, Thermal, Textural, and Rheological Properties of Corn Starch. Foods.

[41] da Silva Costa R.A., Bonomo R.C.F., Rodrigues L.B., Santos L.S., Veloso C.M. (2020). Improvement of texture properties and syneresis of arrowroot (*Maranta arundinacea*) starch gels by using hydrocolloids (guar gum and xanthan gum). J. Sci. Food Agric..

[42] Gunasekara D., Bulathgama A., Wickramasinghe I. (2021). Comparison of Different Hydrocolloids on the Novel Development of Muffins from “Purple Yam” (*Dioscorea alata*) Flour in Sensory, Textural, and Nutritional Aspects. Int. J. Food Sci..

[43] Liu G., Wang J., Hou Y., Huang Y.-B., Wang J., Li C., Guo S., Li L., Hu S.-Q. (2018). Characterization of wheat endoplasmic reticulum oxidoreductin 1 and its application in Chinese steamed bread. Food Chem..

[44] Rathnayake H., Navaratne S., Navaratne C. (2018). Porous crumb structure of leavened baked products. Int. J. Food Sci..

[45] Rojas J.A., Rosell C.M., De Barber C.B. (1999). Pasting properties of different wheat flour-hydrocolloid systems. Food Hydrocoll..

[46] Mohamed A., Hussain S., Alamri M.S., Ibraheem M.A., Qasem A.A.A., Ababtain I.A. (2022). Physicochemical Properties of Starch Binary Mixtures with Cordia and Ziziphus Gums. Processes.

[47] Matia-Merino L., Prieto M., Roman L., Gómez M. (2019). The impact of basil seed gum on native and pregelatinized corn flour and starch gel properties. Food Hydrocoll..

[48] Huang S., Chi C., Li X., Zhang Y., Chen L. (2022). Understanding the structure, digestibility, texture and flavor attributes of rice noodles complexation with xanthan and dodecyl gallate. Food Hydrocoll..

